# Predictors of delayed Anti-Retroviral Therapy initiation among adults referred for HIV treatment in Uganda: a cross-sectional study

**DOI:** 10.1186/s12913-023-09052-z

**Published:** 2023-01-16

**Authors:** Micheal Kiyingi, Joaniter I. Nankabirwa, Christine Sekaggya-Wiltshire, Joan Nangendo, John M. Kiweewa, Anne R. Katahoire, Fred C. Semitala

**Affiliations:** 1 Department of Medicine, Makerere University College of Health Sciences, Makerere University, Box 7062, Kampala, Uganda; 2 Makerere University Joint AIDS Program, Makerere University, Kampala, Uganda; 3 Infectious Diseases Research Collaboration, Kampala, Uganda; 4 Infectious Diseases Institute, Makerere University, Kampala, Uganda; 5 Education Department, Fairfield University, Fairfield, CT USA; 6 Child Health and Development Center, Makerere University, Kampala, Uganda

**Keywords:** Prevalence, Predictors, Persons living with HIV, Anti-retroviral therapy, Initiation, Referral

## Abstract

**Background:**

Uganda’s current guidelines recommend immediate initiation of Anti-Retroviral Therapy (ART) for persons living with HIV in order to reduce HIV/AIDS related morbidity and mortality. However, not all eligible PLHIV initiate ART within the recommended time following HIV diagnosis. We assessed the prevalence and factors associated with delayed ART initiation among PLHIV referred for ART initiation, five years since rolling out the test and treat guidelines.

**Methods:**

In this cross-sectional study, we enrolled adult patients referred to Mulago Immune Suppressive Syndrome (Mulago ISS) clinic for ART initiation from January 2017 to May 2021. We collected data on socio-demographics, HIV diagnosis and referral circumstances, and time to ART initiation using a questionnaire. The outcome of interest was proportion of patients that delayed ART, defined as spending more than 30 days from HIV diagnosis to ART initiation. We performed multivariable logistic regression and identified significant factors.

**Results:**

A total of 312 patients were enrolled of which 62.2% were female. The median (inter-quartile range [IQR]) age and baseline CD4 count of the patients were 35 (28–42) years and 315 (118.8–580.5) cells/μL respectively. Forty-eight (15.4%) patients delayed ART initiation and had a median (IQR) time to ART of 92 (49.0–273.5) days. The factors associated with delayed ART initiation were; 1) having had the HIV diagnosis made from a private health facility versus public, (adjusted odds ratio [aOR] = 2.4 (95% confidence interval [CI] 1.1–5.5); 2) initial denial of positive HIV test results, aOR = 5.4 (95% CI: 2.0–15.0); and, 3) having not received a follow up phone call from the place of HIV diagnosis, aOR = 2.8 (95% CI: 1.2–6.8).

**Conclusion:**

There was significant delay of ART initiation among referred PLHIV within 5 years after the rollout of test and treat guidelines in Uganda. Health system challenges in the continuity of HIV care services negatively affects timely ART initiation among referred PLHIV in Uganda.

## Introduction

In 2015, the World Health Organization (WHO) recommended the test and treat (TAT) strategy to promote early Antiretroviral Therapy (ART) initiation which had been shown to prevent the progression of HIV infection to Acquired Immunodeficiency Syndrome (AIDS), reduce secondary HIV transmission and improve quality of life including life expectancy among People Living with HIV (PLHIV) [1–4]. Through the Uganda Ministry of Health, the government revised the national consolidated guidelines for HIV prevention, treatment and support to include the new recommendation of TAT [5, 6]. These guidelines, first implemented in 2017, recommend that PLHIV to be offered ART at the same health facility within 7 days of a confirmed HIV diagnosis. For patients who are diagnosed with HIV in the community or at health facilities that are different from where they are to receive HIV treatment, ART should be initiated within 30 days of HIV diagnosis [5, 6]. Despite evidence of benefits of early ART initiation among PLHIV, delayed HIV treatment remains a major concern in sub-Saharan Africa (SSA) [7–9]. Most studies on delayed ART initiation in Uganda have been conducted among hospitalized patients [10, 11] reporting over 50% of patients delaying ART, with a CD4 > 50 cells/μL and long distance from health facility associated with delayed ART initiation. Other factors associated with delayed ART initiation found elsewhere were male gender, low education level, older age, opportunistic infections and a high CD4 count [8, 12–15]. Previous studies in SSA show that patients tend to initiate ART at low CD4 counts despite test and treat, and the prevalence of advanced HIV disease is still high [16–20]. In light of the progress made to achieve the Joint United Nations on HIV/AIDS (UNAIDS) target of 90-90-90 [21, 22] in Uganda, we hypothesized that some patients who are referred may be lost to follow up despite knowing their HIV status and present to HIV treatment centers later on. We thought this could be due to challenges in the health system structure in addition to individual and disease factors. Therefore, we sought to investigate the prevalence and factors associated with delayed ART initiation among PLHIV referred to Mulago ISS clinic in the era of test and treat.

### HIV and the health care system in Uganda

According to the national consolidated guidelines of HIV prevention, treatment and support, the entry point into the HIV care continuum [23] is through an HIV test, which can be performed at a private, private not-for-profit (PNFP), or public health facility, during community outreach activities or a self-test [6]. If the test is negative, the individual is offered HIV prevention services. If the test is positive, the patient is linked to care in any of the many HIV clinics in the country. The HIV clinics are located in public and PNFP health facilities only, and HIV care programs support their operations. These programs comprehensively implement the HIV care continuum, in collaboration with the ministry of health, including provision of free ART medicines. Hence, most privately owned health facilities do not have access to ART medicines but provide HIV testing services. Therefore, there are newly diagnosed PLHIV who are tested in private health facilities, in community outreaches and some public health facilities who will need to be referred to another health facility to access free ART services. Despite this need for referral and availability of standard referral notes [24], only draft 2017 national referral guidelines exist in Uganda and the practice varies from place to place [25]. Therefore, not all patients are written referral notes or have follow up phone calls made to ascertain completion of the process. Patients can walk into any HIV clinic with or without referral documents and receive care. However, prior to reception at the HIV clinic, these patients are not known as PLHIV and it is urgent that they reach the clinic as early as possible.

## Methods

### Design and setting

This cross sectional study was conducted at Mulago-ISS clinic. Established in 2007, the Mulago ISS clinic is a center of excellence for comprehensive HIV and Tuberculosis (TB) services, and it is the largest HIV clinic in Uganda, serving a population of over 16,500 PLHIV. This clinic is located in Mulago hospital Complex, the largest tertiary hospital in Uganda located in the capital city called Kampala. The clinic receives patients referred from Kampala and neighboring districts. All services at this clinic are supported by the Makerere University Joint AIDS Program (MJAP), and are offered at no cost to the patient.

### Study participants and data collection

We consecutively screened and enrolled adult patients who presented to the Mulago ISS clinic between November 2020 and May 2021. Patients were included in the study if they were: 1) aged 18 years and above; 2) diagnosed with HIV in 2017 or later, outside of Mulago ISS clinic; 3) referred to Mulago ISS clinic and initiated on ART in 2017 or later; 4) provided written informed consent to participate in the study.

Data was collected from patients and their records using an interviewer-administered questionnaire. From patients, we collected data on the demographics including their gender, age, socio-economic status, marital status and highest level of education. In addition, we also collected data on the place and date of HIV diagnosis, whether they received counselling during testing, if they immediately accepted HIV results, if they were given referral notes and/or received follow up phone calls after referral for ART initiation. From the patients’ records, we collected data on dates of ART initiation, clinical data at the time of presentation for ART including the baseline CD4 count, a diagnosis of tuberculosis and/or cryptococcal meningitis prior to ART initiation. Immediate ART initiation is not recommended if a patient has either tuberculosis or cryptococcal meningitis [6, 26–28]. Delayed ART initiation was defined as more than 30 days elapsing between date of HIV diagnosis and date of ART initiation.

### Statistical analysis

Data was entered into Microsoft Excel and analyses were performed using Stata, version 14 (Stata Corporation, College Station, Texas, USA). Data on missing baseline CD4 counts were resolved by multiple imputation method. Baseline characteristics of the patients were summarized as percentages if categorical and as medians with their interquartile ranges (IQR) if continuous. Principal component analysis was used to estimate the social economic status and was presented as pentiles [29]. The primary outcome was the prevalence of delayed ART initiation, which was defined as having more than 30 days elapse between HIV diagnosis and ART initiation. Both simple binary and multivariable logistic regression were performed to identify significant factors. A total of 8 factors were fitted in the multivariable model based on a frequency of five or more and/or a *p*-value of ≤0.2 at simple binary regression. The model goodness of fit was assessed using the Hosmer – Lemeshow test (*p* = 0.08). A backward elimination method was used by removing the least likelihood variables until only significant factors emerged. Odds ratios with their 95% confidence interval and *p*-values are presented. A *p*-value of ≤0.05 was considered significant.

## Results

### Characteristics of the study population

Between November 2020 and May 2021, 450 patients were screened for eligibility and 312 (69.3%) were enrolled. Reasons for exclusion of the 138 patients included decline to provide consent for the study 24 (17.4%) and lacked information on date of HIV diagnosis 114 (82.6%). Four of the 312 included in the final analysis had missing baseline CD4 count, which we resolved. The median (IQR) age of the 312 patients enrolled was 35 (28–42) years and CD4 count was 315 (118.8–580.5) cells/μL. More than half, 182 (58.3%), had been diagnosed with HIV in a public health facility, 246 (78.9%) were given a referral note and 189 (60.6%) received a follow up phone call from the place of HIV diagnosis to establish if they had reached the referral center (Table 1).

**Table 1:** Characteristics of PLHIV received as referrals for ART initiation at Mulago ISS clinic in the test and treat era (*N* = 312)

Characteristic	Frequency, n (%)
Age group	
18–24	23 (7.4)
25–34	129 (41.3)
> =35	160 (51.3)
Sex	
Male	118 (37.8)
Female	194 (62.2)
Marital status	
Single	172 (55.1)
Married	140 (44.9)
Socioeconomic status	
Wealthiest	15 (4.8)
Wealthy	108 (34.6)
Medium	52 (16.7)
Poor	51 (16.4)
Poorest	86 (27.6)
Highest level of education	
None/primary	149 (47.8)
Secondary	125 (40.1)
Tertiary	38 (12.2)
Employment status	
Not employed	54 (17.3)
Self employed	166 (53.2)
Formal employment	92 (29.5)
Place of HIV diagnosis	
Public health facility	182 (58.3)
Private health facility	103 (33.0)
Community outreach	25 (8.0)
Self-test	2 (0.6)
Counselling during HIV positive test	
None	25 (8.0)
Either pre or post testing	48 (15.4)
Both pre and post testing	239 (76.6)
Accepted HIV positive results	
Yes	283 (90.7)
No	29 (9.3)
Given a referral form	
Yes	246 (78.9)
No	66 (21.2)
Received a follow-up phone call from place of diagnosis	
Yes	189 (60.6)
No	123 (39.4)
Baseline CD4 count	
> =200 cells/μL	208 (66.7)
< 200 cells/μL	104 (33.3)
HIV associated comorbidities	
None	279 (89.4)
Tuberculosis	28 (9.0)
Cryptococcal meningitis	5 (1.6)

### Prevalence of delayed ART initiation

Of the 312 patients enrolled, 48 (15.4%) delayed to initiate ART, with the median (IQR) time to ART among those with delayed initiation of 92 (49–273.5) days. Among patients with delayed ART initiation, 62.5% were females, median (IQR) age was 33 (27–45) years, and median (IQR) CD4 count was 342 (186.5–566.5) cells/μL. About one in three [17 (35.4%)] of these belonged to the poorest quintile, and 25 (52.1%) had their HIV diagnosis made from a private health facility.

### Factors associated with delayed ART initiation

The factors significantly associated with delayed ART initiation included 1) the health center/setting where the HIV diagnosis was made, 2) acceptance of the HIV results at the time of testing HIV positive and 3) receiving a follow up call to confirm/remind the patient to report to a higher health facility for ART initiation from the facility where they tested HIV positive. Patients diagnosed with HIV at private health facilities had significantly higher odds of delayed ART initiation compared to those diagnosed at public health facilities (adjusted odds ratio [aOR] = 2.4, 95% Confidence Interval [CI] 1.1–5.5, *p* = 0.037). Similarly, patients who initially denied their HIV positive test results at the time of HIV diagnosis had higher odds of delaying ART than those that accepted their results (aOR = 5.4, 95% CI: 2.0–15.0, *p* = 0.001). Finally, patients who did not receive a follow up phone call after referral had higher odds of delayed ART initiation compared to those that received a call (aOR = 2.8, 95% CI: 1.2–6.8, *p* = 0.019) (Table 2).

**Table 2:** Factors associated with of delayed ART initiation among PLHIV referred for ART at Mulago ISS clinic in the test and treat era

Variable	Category	***n*** = 48 (%)	cOR (95% CI)	***P***-value	aOR (95% CI)	***P***-value
Age group	15–24	4 (8.3)	1.0			
	25–34	22 (45.8)	0.8 (0.3–2.5)	0.730		
	> = 35	22 (45.8)	1.3 (0.3–5.4)	0.765		
Sex	Female	30 (62.5)	1.0			
	Male	18 (37.5)	1.0 (0.5–1.9)	0.960		
Highest level of Education	None/primary	21 (43.8)	1.0		1.0	
	Secondary	17 (35.4)	1.0 (0.5–1.9)	0.906	0.9 (0.4–2.0)	0.771
	Tertiary	10 (20.8)	2.2 (0.9–5.1)	0.075	2.4 (0.8–7.1)	0.116
Employment status	Not employed	4 (8.3)	1.0			
	Self employed	27 (56.3)	2.4 (0.8–7.3)	0.114		
	Formal employment	17 (35.4)	2.8 (0.9–8.9)	0.075		
Marital status	Single	26 (54.2)	1.0			
	Married	22 (45.8)	1.0 (0.6–1.9)	0.884		
SES	Poorest	17 (35.4)	1.0		1.0	
	Poor	6 (12.5)	0.5 (0.2–1.5)	0.230	0.4 (0.1–1.5)	0.173
	Medium	5 (10.4)	0.4 (0.1–1.3)	0.122	0.4 (0.1–1.2)	0.101
	Wealthy	14 (29.2)	0.6 (0.3–1.3)	0.202	0.5 (0.2–1.3)	0.150
	Wealthiest	6 (12.5)	1.0 2.7 (0.8–8.6)	0.093	2.5 (0.6–10.5)	0.211
Place of HIV diagnosis	Public facility	17 (35.4)	1.0		1.0	
	Private facility	25 (52.1)	3.1 (1.6–6.1)	**0.001**	2.4 (1.1–5.5)	**0.037**
	Outreach	6 (12.5)	2.4 (0.8–7.3)	0.114	2.9 (0.8–10.4)	0.096
Counselling during HIV diagnosis	Both pre and post testing	28 (58.3)	1.0		1.0	
	Either pre or post testing	10 (20.8)	2 (0.9–4.4)	0.086	1.0 (0.4–2.5)	0.933
	None	10 (20.8)	5 (2.1–12.3)	**< 0.001**	1.3 (0.4–4.3)	0.710
Accepted HIV positive results	Yes	33 (68.8)	1.0		1.0	
	No	15 (31.3)	8.1 (3.6–18.3)	**< 0.001**	5.4 (2.0–15.0)	**0.001**
Received a referral note to higher health facility	Yes	24 (50.0)	1.0		1.0	
	No	24 (50.0)	5.3 (2.7–10.2)	**< 0.001**	1.7 (0.7–4.5)	0.244
Received a follow-up phone call after referral from place of testing.	Yes	15 (31.3)	1.0		1.0	
	No	33 (68.8)	4.3 (2.2–8.2)	**< 0.001**	2.8 (1.2–6.8)	**0.019**
Baseline CD4 count before ART initiation.	< 200 cells/μL	13 (27.1)	1.0		1.0	
	≥200 cells/μL	35 (72.9)	1.4 (0.7–2.8)	0.320	1.3 (0.6–2.9)	0.515
HIV associated comorbidities	None	44 (91.7)	1.0			
	Tuberculosis	1 (2.1)	0.2 (0.03–1.5)	0.116		
	Cryptococcal Meningitis	3 (6.3)	8.0 (1.3–49.3)	**0.025**		

*n* Number that delayed ART initiation, *cOR* Crude Odds Ratio, *aOR* Adjusted Odds Ratio, *SES* Social economic status

## Discussion

This study assessed the prevalence and factors associated with delayed ART initiation among HIV positive patients referred for ART initiation. We found a high prevalence of delayed ART initiation with one in every six referred PLHIV experiencing delay in ART initiation. The median duration between HIV diagnosis and ART initiation among PLHIV who delayed ART initiation was up to 92 days. These findings provide insights into delays in ART initiation for patients referred to HIV treatment centers from facilities that provide HIV testing but no treatment services. Based on the national estimates, 13% of HIV positive patients who know their status were not on ART [6] which is higher than the 2020 target of at most 10% [21]. Therefore, there is urgent need to accelerate efforts to link newly diagnosed PLHIV into ART programs as soon as possible, in order to achieve the ‘95% PLHIV initiated on ART’ target by 2030 [30].

Among the factors significantly associated with delayed ART initiation, we found that patients who had their HIV diagnosis made from private health facilities had significantly higher odds of delayed ART initiation compared to those diagnosed in public health facilities. This finding reflects the health system challenges private health facilities in Uganda face to provide comprehensive HIV care services. Despite providing HIV testing services, private health facilities do not have access to free ART for patients and are not directly supported by the HIV care programs as are public health facilities. With the absence of harmonized national referral guidelines, these health facilities face a challenge of continuity of care after an HIV test and contribute to late presentation and delayed ART initiation. An assessment of the provision of HIV care in the private health facilities can help identify, bridge gaps and expedite provision of services at each opportunity for PLHIV.

Still at health system level, during referral for ART, we found that patients who did not receive follow up phone calls from the place of HIV diagnosis had higher odds of delayed ART initiation as compared to patients who received. This also reflects a challenge in continuity of care after a positive HIV test for patients who are referred. Despite the presence of standard referral forms, the lack of guidelines makes the referral practice vary, with some health facilities adding follow up phone calls to ascertain completion of referral process and others not. Due to the urgency needed to provide early ART in PLHIV, harmonized referral guidelines including follow up phone calls are needed to link more patients into care and end AIDS by 2030. Evidence from settings similar to Uganda have shown that clear referral procedures including follow up phone calls can facilitate early initiation of ART at a health facility [31–33].

At individual level, we found that immediate denial of HIV positive results was also significantly associated with delayed ART initiation among patients referred for ART. This may be related to the quality of counselling and support given to PLHIV from the different HIV testing settings. Previous studies in Uganda and SSA have shown that denial of HIV status is associated with delayed ART initiation [34, 35], while early acceptance is associated with timely initiation [36]. Larger studies investigating linkage to care from different testing settings are needed to study this phenomenon further. On the other hand, we did not find statistical significance with other individual factors like sex, age and socio-economic status which have been found to affect ART initiation in most other studies previously conducted in SSA [12, 13, 37]. This may be because we studied a population with very limited information in our setting.

### Strengths

The study was conducted at a center of excellence which has good data practices, a large sample of patients to choose from and operational and well supported systems. The population studied was not specifically studied before, which provides a benchmark for interventions and further research.

### Limitations

Because this study was conducted at a center of excellence in comprehensive HIV services, the prevalence of delayed ART initiation among referred patients may be higher in the HIV clinics at the lower level health centers that have much more patients combined with weaker and poorly supported systems.

Despite an adequate sample size, there was a relatively small number of patients who delayed ART initiation and a high variance in the data which negatively affected the power of this study. A larger study at multiple HIV clinics would address this.

## Conclusion

There has been a general improvement in the timing of ART initiation following a positive HIV test in Uganda. However, 15% of HIV positive patients referred for ART delayed to initiate ART despite testing positive within 5 years after the test and treat guidelines were rolled out. Health system challenges still exist and they negatively affect timely ART initiation among referred PLHIV in Uganda. Interventions to ensure timely continuity of care along the HIV care continuum would facilitate early ART initiation among referred PLHIV.

## Data Availability

The datasets generated and analyzed for this study are not publicly available because medical file numbers were used on patients’ questionnaires to avoid re-interviewing the same participant. However, these are available from the corresponding author upon reasonable request.

## Abbreviations

ART: Anti-retroviral Therapy
HIV: Human Immunodeficiency virus
PLHIV: People Living With HIV
UNAIDS: Joint United Nations Program on HIV/AIDS
WHO: World Health Organization
